# A conserved R type Methionine Sulfoxide Reductase reverses oxidized GrpEL1/Mge1 to regulate Hsp70 chaperone cycle

**DOI:** 10.1038/s41598-018-21083-9

**Published:** 2018-02-09

**Authors:** Praveen Kumar Allu, Yerranna Boggula, Srinivasu Karri, Adinarayana Marada, Thanuja Krishnamoorthy, Naresh Babu V. Sepuri

**Affiliations:** Department of Biochemistry, School of Life Sciences, University of Hyderabad, Gachibowli, Hyderabad, 500046 India

## Abstract

Cells across evolution employ reversible oxidative modification of methionine and cysteine amino acids within proteins to regulate responses to redox stress. Previously we have shown that mitochondrial localized methionine sulfoxide reductase (Mxr2) reversibly regulates oxidized yeast Mge1 (yMge1), a co-chaperone of Hsp70/Ssc1 to maintain protein homeostasis during oxidative stress. However, the specificity and the conservation of the reversible methionine oxidation mechanism in higher eukaryotes is debatable as human GrpEL1 (hGrpEL1) unlike its homolog yMge1 harbors two methionine residues and multiple cysteines besides the mammalian mitochondria hosting R and S types of Mxrs/Msrs. In this study, using yeast as a surrogate system, we show that h*GRPEL1* and R type *MSRs* but not the S type *MSRs* complement the deletion of yeast *MGE1* or *MXR2* respectively. Our investigations show that R type Msrs interact selectively with oxidized hGrpEL1/yMge1 in an oxidative stress dependent manner, reduce the conserved hGrpEL1-Met146-SO and rescue the Hsp70 ATPase activity. In addition, a single point mutation in hGrpEL1-M146L rescues the slow growth phenotype of yeast *MXR2* deletion under oxidative duress. Our study illustrates the evolutionarily conserved formation of specific Met-R-SO in hGrpEL1/yMge1 and the essential and canonical role of R type Msrs/Mxrs in mitochondrial redox mechanism.

## Introduction

Redox imbalance is one of the key factors for myriad of diseases including metabolic and neurodegenerative disorders^1,2^. Enhanced levels of Reactive Oxygen Species (ROS) or alterations in antioxidant mechanisms affect the activities of biomolecules with reversible and irreversible modifications. Interestingly, ROS also has a beneficial side to it as they known to play as signaling molecule for various cellular functions including redox regulation of several transcription factors, kinases and enzymes^3,4^. Protein reversible oxidative modification, in particular at cysteine and methionine amino acid residues play an important role in many cellular functions. Methionine, upon oxidation forms reversible diastereomeric methionine sulfoxide (Met-S-SO or Met-R- SO) and these sulfoxides are specifically reduced by methionine sulfoxide reductase MsrA or MsrB respectively in a thioredoxin dependent mechanism^5,6^. Deletion of *MSRs* is known to enhance cellular ROS levels that have been implicated in several diseases including diabetes, neurodegeneration, and aging^7^. Methionine in proteins is known to act as an antioxidant and regulate several proteins that undergo Met-SO dependent structural destabilization. Recent studies predict that methionine oxidation could act like protein phosphorylation in cellular signaling to regulate several protein functions^8,9^.

Chaperones play an important role in mitochondrial biogenesis through an efficient protein translocation, assembly, iron-sulfur cluster formation, mtDNA maintenance and protein homeostasis^10^. The indispensable Hsp70 chaperone system consists of several conserved components that include DnaK/DnaJ or J-complex and Mge1/GrpE proteins. Mge1/GrpEL1, a conserved nucleotide exchange factor, in its dimeric form interacts with Hsp70-ADP-substrate complex to facilitate the exchange of ADP for ATP so as to initiate another round of Hsp70 cycle. Consequently, it has been shown that GrpE enhances DnaK ATPase cycle by 5000 folds^11^. The stoichiometry between GrpE orthologs and Hsp70/DnaK is shown to be 2:1^12,13^. Mge1/GrpE protein in Hsp70 chaperone system can undergo early stress dependent structural transition. Oxidative and thermal stresses are known to change the ratio between active dimeric Mge1 to inactive monomeric form^14,15^. We have shown earlier that the conserved methionine at 155^th^ position in Mge1 responds to oxidative stress. In addition, mitochondrial localized methionine sulfoxide reductase 2 (Mxr2) reversibly regulates Mge1 by selectively reducing the Met155-SO to restore the activity of Mge1. Although, Mxr2 reduces the Met-SO of yeast Mge1 both *in vitro* and *in vivo*, our earlier study does not preclude the formation of only R type sulfoxide upon oxidation.

In contrast to yeast, mammalian mitochondria contain two isoforms of R type (MsrB2 and MsrB3) and one isoform of S type (MsrA) sulfoxide reductases^5,6^. In addition, two isoforms of GrpEL (GrpEL1 and GrpEL2) are present in mammalian mitochondria. GrpEL1 and GrpEL2 are differentially expressed across all tissues and high levels of GrpEL1 protein has been detected in many tissues^16,17^. To further increase the complexity, mammalian GrpEL1 contains multiple cysteines and two methionine residues (position 44 and 146) while its counterpart in yeast, Mge1 lacks cysteines and contains only one methionine (position 155). It is remains to be explored whether the MsrB mediated redox switch at conserved GrpEL1 methionine residue is required for GrpEL1 function and subsequent regulation of Hsp70/Ssc1p cycle.

In this study, we show that both the methionine residues in human GrpEL1 get oxidized upon exposure to H_2_O_2_. However, the conserved oxidized methionine at 146 is specifically reduced by R type of methionine sulfoxide reductase *in vitro*. Using an yeast heterologous system, we show that human *hGRPEL1* and human R type *MSR* complement the deletion of yeast *MGE1* and *MXR2* respectively. Yeast cells expressing human GrpEL1-M146L mutant conferred better growth kinetics than yeast strain expressing wild type hGrpEL1 under oxidative stress. This study delineates the function of human GrpEL1 and R type Msrs in redox regulation besides the evolutionarily conserved role of Mge1/GrpEL1 in mitochondrial oxidative stress response pathway.

## Results

### hGrpEL1 responds to oxidative stress and alters the ATPase stimulating activity of Hsp70/Ssc1 *in vitro*

**Figure 1:**
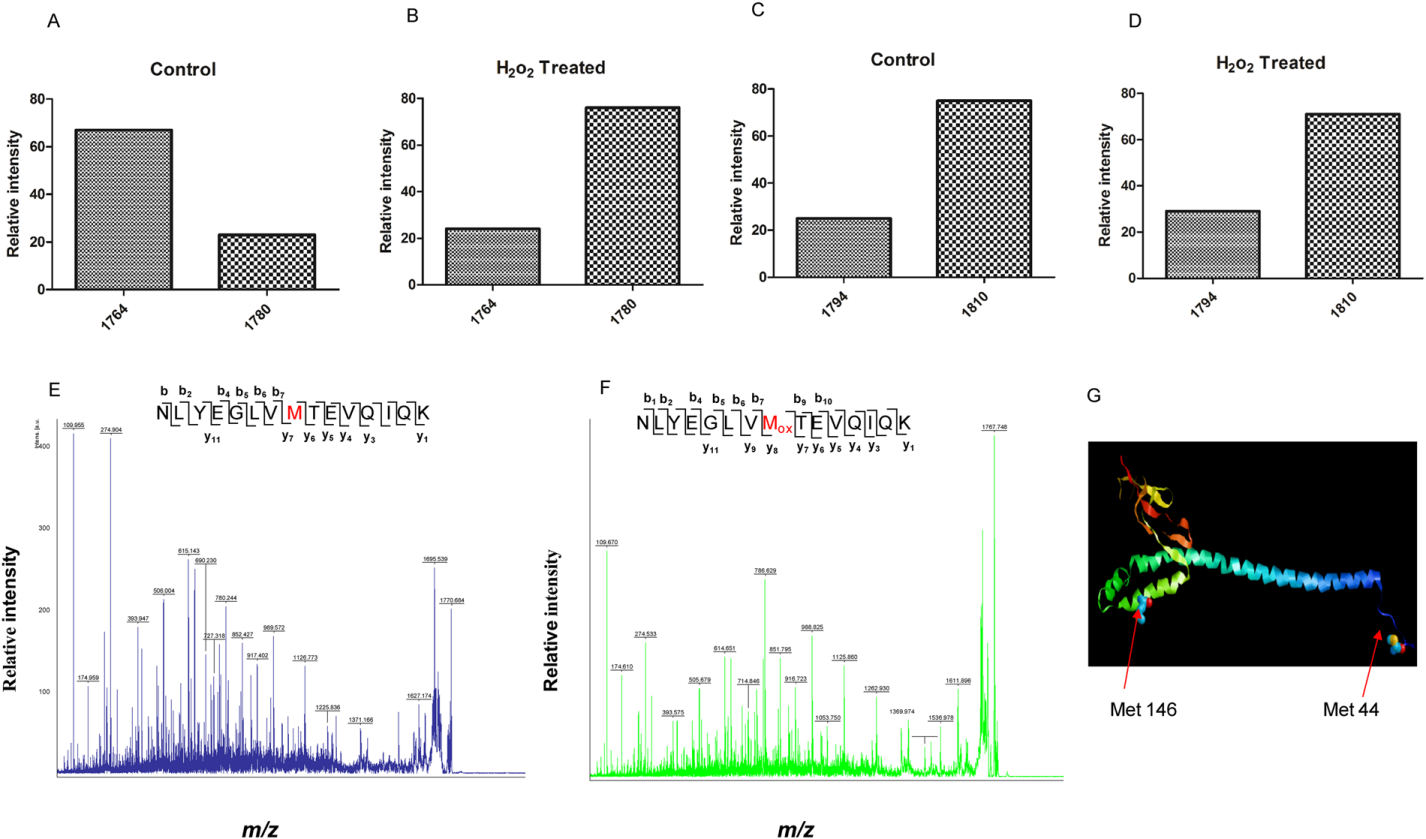
Oxidation of Met146 in hGrpEL1 *in vitro*. Purified human His-GrpEL1 recombinant protein was treated with or without H_2_O_2_, separated on SDS-PAGE and the gel Coomassie stained. The GrpEL1 protein bands were excised from the gel and trypsin digested. The resulting peptides were analyzed by MALDI-TOF/MS/MS. From the MALDI spectra, the intensity of the peaks was measured and taken as a percentage against the total intensity of oxidized and un-oxidized peptides. (**A**) and (**B**) MALDI spectra showing percentage of oxidized (1764 Da) and un-oxidized (1780 Da) Met146 of hGrpEL1. (**E**) and (**F**) Peptides 1764 Da and 1780 Da from control (Fig. 1A) and H_2_O_2_ treated hGrpEL1 (Fig. 1B) were MS/MS sequenced and analyzed. (**C**) and (**D**) Percentage of oxidized (1794 Da) and un-oxidized (1810 Da) Met44 from MALDI spectra of H_2_O_2_ treated hGrpEL1 (Fig. 1B). The relative levels of total oxidized and un-oxidized M146 and M46 peptides were calculated from three independent experiments and the average of total oxidized and un-oxidized peptides are shown. (**G**) Representation of methionine’s in hGrpEL1 by I TASSER GrpEL1 structure and space fill molecules.

Mge1 is known to be oxidized at conserved Met155 amino acid residue both *in vitro* and *in vivo* upon exposure to oxidative stress. Human GrpEL1 contains methionine residue at 44^th^ and 146^th^ position and the latter one is analogous to yMge1 Met155. To test whether hGrpEL1 is oxidized at conserved methionine residue like yMge1, purified recombinant hGrpEL1 was treated with or without H_2_O_2_, separated on SDS-PAGE, Coomassie stained and trypsin digested fragments were analyzed by MALDI-TOF-MS/MS (Supplementary Figure S2A–D). In the absence of H_2_O_2_, Met146 containing peptides were resolved as a major un-oxidized 1764 Da mass and as a minor oxidized 1780 Da mass with a difference of 16 Da (Fig. 1A). In contrast, H_2_O_2_ treated hGrpEL1 displayed a relatively higher form of oxidized Met146 peptide compared to the un-oxidized Met146 peptide (Fig. 1B). MS/MS analysis of the aforementioned peptides confirmed that Met also exists as Met-SO by attaining a mass of 16 Da with H_2_O_2_ treatment (Fig. E and F). Examination of the Met44 containing peptides revealed that Met44 also exists in two forms, the minor un-oxidized 1794 Da form and the major oxidized 1810 form, the latter becoming the more dominant form in the presence of H_2_O_2_ (Fig. 1C and D). Interestingly, Met44 amino acid is located in un-structured N-terminal region whereas Met146 residue is present in two helix bundle domain of Mge1 (Fig. 1G).

**Figure 2:**
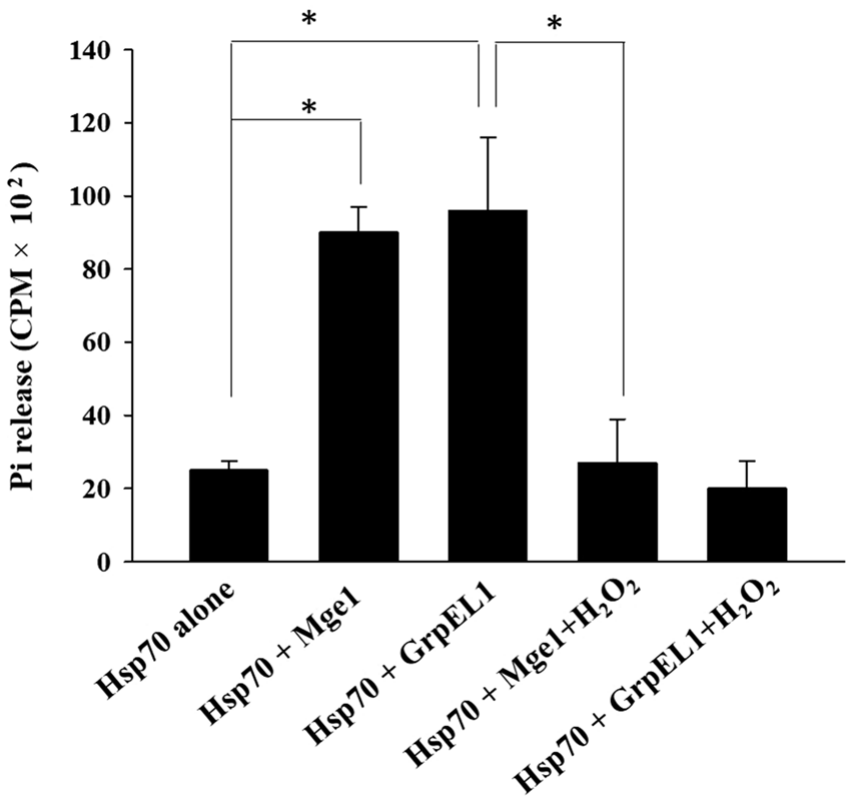
hGrpEL1 promotes yHsp70/Ssc1 ATPase cycle *in vitro*. ATPase activity of recombinant yHsp70/Ssc1 was performed as described in the Methods section. Radioactive ^32^P counts from three experiments were measured using scintillation counter and plotted. The values are mean values obtained from three technical replicates. Each value is mean ± SE (n = 3), *p ≤ 0.05.

To test the significance of oxidized hGrpEL1, we performed an ATPase activity of purified yeast mitochondrial His-yHsp70/Ssc1in the presence of Mge1 or hGrpEL1 with or without H_2_O_2_ treatment as described in the methods. In the absence of GrpEL1/Mge1 proteins, the total yHsp70/Ssc1 mediated ATP hydrolysis in the reaction is minimal (Fig. 2). In the presence of yMge1 or hGrpEL1, the ATPase activity of yHsp70/Ssc1 is enhanced by 3–4 folds. However, the ATPase activity of yHsp70/Ssc1 is reduced when yMge1 or hGrpEL1 was treated with H_2_O_2_ prior to its addition in the assay (Fig. 2). This result suggests that oxidation of GrpEL1 protein might have physiological consequences on the chaperone activity of yHsp70/Ssc1.

### *hGRPEL1* complements the deletion of yeast *MGE1*

**Figure 3:**
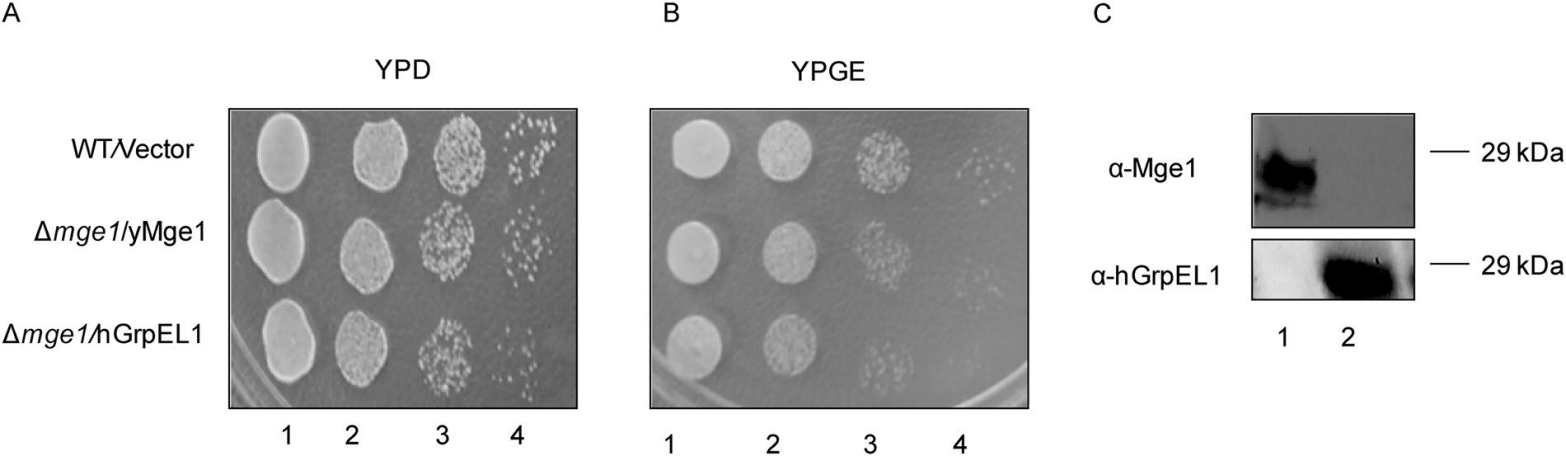
*hGRPEL1* complements yeast *MGE1* deletion. **(A**,**B**) Yeast strains expressing WT yMge1 and hGrpEL1 were grown overnight in YPD, normalized to OD_600_ 0.5 and 10 µl of each dilution was spotted on YPD (**A**) and YPGE (**B**) plates. (**C**) Immunoblot of Whole Cell Extracts (WCE) from yeast strains over-expressing yMge1 (lane 1), WT hGrpEL1 (lane 2) using antibodies against yMge1 (top panel) and hGrpEL1 (lower panel) is shown.

Mge1 is an essential protein and it has been shown that human *GRPEL1* does not complement the deletion of *MGE1* deletion^18^. To investigate if the function of *MGE1* is conserved across evolution, we initially examined if over-expression of h*GRPEL1* could complement *yMGE1* chromosomal deletion. Using plasmid shuffling, we created yeast strains deleted for chromosomal *MGE1* but over-expressing *yMGE1* or *hGRPEL1* from a high copy plasmid as described in the methods^19^. The growth phenotypes of strains expressing *yMGE1* and *hGRPEL1* were compared on YPD and YPGE plates. Strain expressing *hGRPEL1* had growth that was comparable to the strain expressing *yMGE1* on YPD and YPGE plates at 30 °C (Fig. 3A and B). To ensure the protein expression of *hGRPEL1*, we carried out immunoblot analysis of cell lysates obtained from strains expressing *yMGE1* and *hGRPEL1* as described in the Methods section. Immunoblotting studies using antibodies specific against Mge1 and hGrpEL1 confirmed the presence of Mge1 and GrpEL1 (Fig. 3C). Mge1 was not detected in the strain expressing *hGRPEL1* confirming that the strain indeed had chromosomal deletion of *MGE1* (Fig. 3C). Our results clearly demonstrate that over expression of *hGRPEL1* can efficiently complement *yMGE1* deletion.

### Human Methionine R sulfoxide Reductase complements yeast *MXR2* deletion and interacts with oxidized hGrpEL1

Oxidation of methionine can result in two forms of enantiomers, Met-R-SO or Met-S-SO. The protein bound Met-SO enantiomers are known to be specifically reduced by either MsrA or MsrB enzymes^20,21^. Mammalian mitochondria contain three methionine sulfoxide reductases that include two R type (MsrB2 and MsrB3) and one S type (MsrA) sulfoxide reductases. As hGrpEL1 is sensitive to oxidative stress *in vitro*, we wished to examine if the Met SO modification is reversible, akin to Mge1.

**Figure 4:**
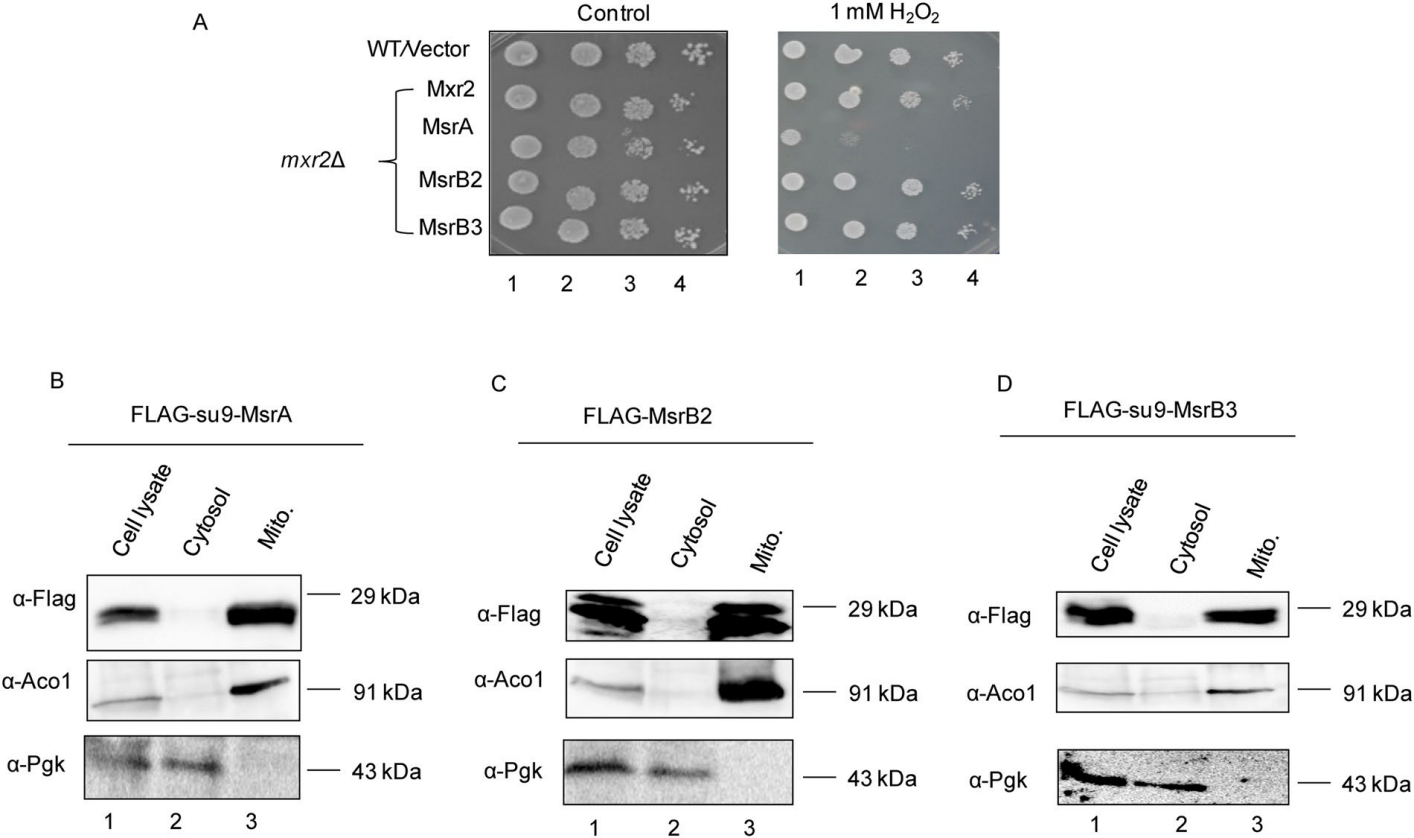
Human R type *MSRs* complement the deletion of yeast *MXR2*. (**A**) Yeast strains expressing Flag-yMxr2, Flag-Su9-hMsrA, Flag-Su9-hMsrB3 and Flag-hMsrB2 respectively were grown overnight in SC-URA medium, normalized, serially diluted and 10 µl of each dilution were spotted on SC-URA plates with or without H_2_O_2_. (**B**–**D**) Yeast strains expressing human Flag-Su9-hMsrA (**B**), Flag-hMsrB2 (**C**) and Flag-Su9-hMsrB3 (**D**) were sub-fractionated as cytosol (lane 2) and mitochondrial fraction (lane 3), separated on SDS-PAGE along with total cell extract (lane 1), blotted and probed with antibodies specific for Flag, cytosolic Pgk1 and mitochondrial AcoI.

It has been shown that mammalian *MSRB* can complement the deletion of yeast *MXR2*^22^. Initially, we analyzed the importance of mitochondrial localized mammalian Msrs during oxidative stress by specifically targeting them to yeast mitochondria. We employed mitochondrial pre-sequence, N-terminal Su-9 MTS, upstream of MSRB3 and MSRA gene sequence to target them to mitochondria. We constructed yeast strains as described in the methods for the expression of human mitochondrial methionine sulfoxide reductases, Su9-MTS-MsrA, Su9-MTS-MsrB3, MsrB2 and yeast Mxr2 with Flag epitopes in a *mxr2*Δ background. The parent strain, WT was transformed with an empty vector and used as a control strain. We compared the growth of Flag-Msr expressing strains with the control strain on SC-URA in the presence and absence of H_2_O_2_. All the strains had comparable growth on SC-URA  plates in the absence of H_2_O_2_. However, in the presence of H_2_O_2_, strains expressing human MsrB2 or MsrB3 or yeast Mxr2 had growth phenotype similar to control strain (Fig. 4A). However, growth of strain expressing mammalian MsrA is severely hampered in the presence of H_2_O_2_. These results suggest that only Met-R-SO reducing enzymes can efficiently complement the *MXR2* deletion. To confirm the mitochondrial localization of the human mitochondrial Msrs in yeast, subcellular fractionation of yeast strains expressing different Flag-Msr was carried out and the fractions were resolved on SDS-PAGE, blotted and probed with antibodies specific to the mitochondria fraction (Aconitase), cytosolic faction (Glycerol kinase) and to the Flag epitope. As shown in the Fig. 4, Flag-Msr proteins are enriched in the mitochondrial fraction like Aconitase (Fig. 4B–D). These results suggest that human Flag-Msr proteins are efficiently targeted to the yeast mitochondria and only the R type of human Msrs are able to complement the deletion of yeast *MXR2*.

**Figure 5:**
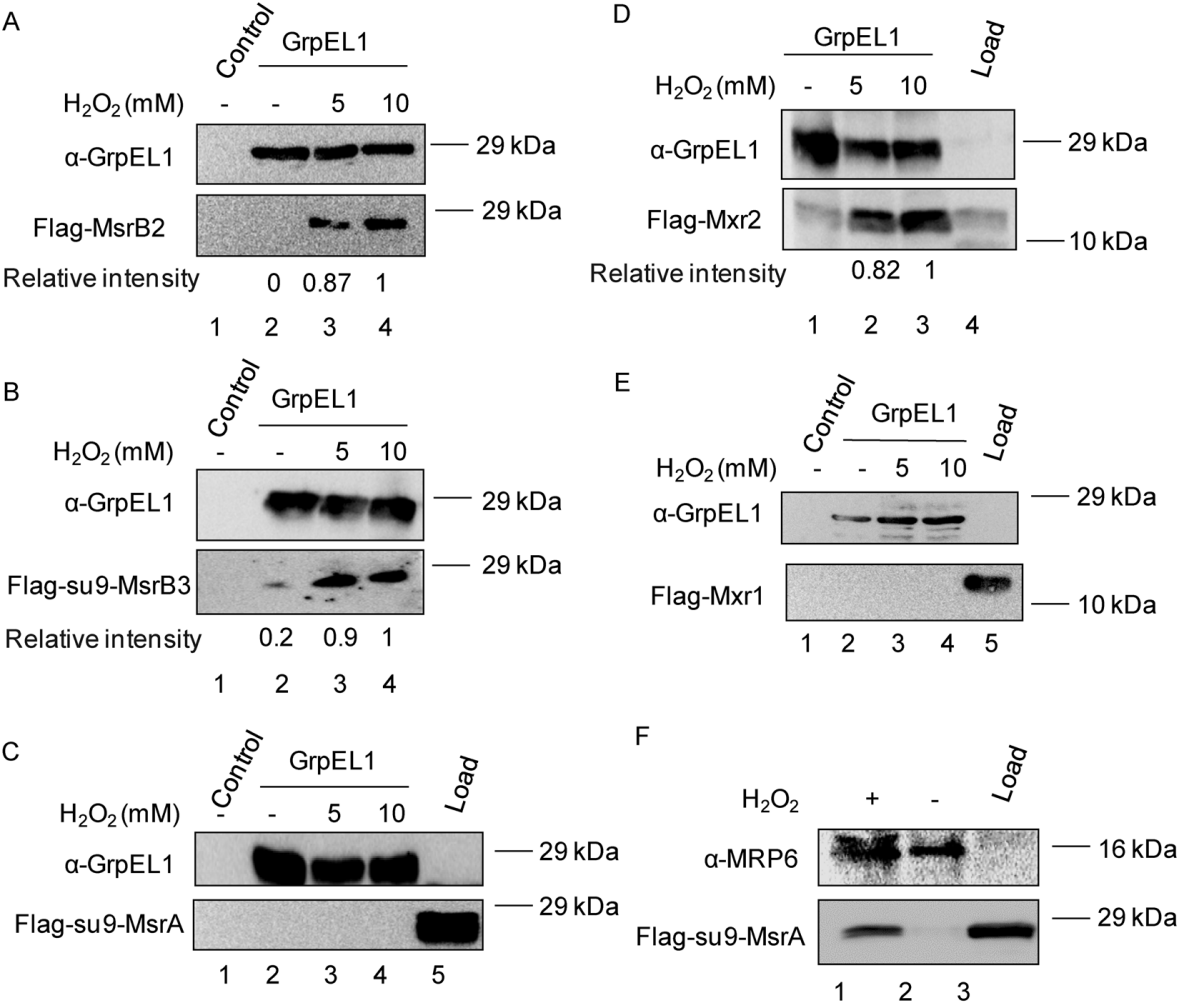
hGrpEL1 interacts with R type sulfoxide reductases in an oxidative stress dependent manner. Purified recombinant His-hGrpEL1 (10 µg) was pretreated without or with increasing concentrations of H_2_O_2_ for 2 hrs prior to binding to Ni-NTA beads. Mitochondrial lysate (100 µg) isolated from strains expressing (**A**) Flag-MsrB2, (**B**) Flag-Su9-MsrB3, (**C**) Flag-Su9-MsrA, (**D**) Flag-yMxr2 or cell extract (100 µg) isolated from strain expressing (**E**) Flag-yMxr1 were allowed to bind to in the absence or presence His-hGrpEL1 pre-bound to Ni-NTA beads as described in the Methods section. Total eluates were resolved on SDS-PAGE and immunoblotted with specific antibodies. Load indicates 20% of the cytosolic extract (Panel C and E) or 10% of mitochondrial extract (Panel D) (**F**) For control experiment, His-Mrp6 protein, a known substrate of MsrA and MsrB was treated with (lane 2) or without H_2_O_2_ (lane 1) prior to binding to Ni-NTA beads. Mitochondrial lysate of yeast strain expressing Flag-MsrA was allowed to bind to His-Mrp6 and the eluate processed as described above. Load indicates the 20% of the cytosolic extract. Relative intensity of Msr protein bound to GrpEL1 was quantified using Image J software and shown (Panel A, B, D).

To determine the specificity of methionine sulfoxide enantiomers, we performed interaction studies between oxidized GrpEL1 and Msr proteins by *in vitro* Ni-NTA pull down assay as described^19^. The cell lysates expressing mammalian and yeast Flag-Msr proteins were passed through Ni-NTA beads pre-bound with recombinant His-GrpEL1 pre-treated with increasing concentrations of H_2_O_2_. To ascertain specificity, only Ni-NTA beads without His-GrpEL1 was used as control. The beads were washed and the proteins bound to Ni-NTA beads were resolved on SDS-PAGE and blotted as described in the Methods section (Fig. 5). Immunoblotting was carried out using antibodies specific to Flag, GrpEL1 and His tag. Our results show that binding of MsrB2, MsrB3 and Mxr2 depends on the oxidation of GrpEL1 (Fig. 5A,B and D). Most importantly, in the absence of H_2_O_2_, the binding of GrpEL1 to the Msrs or Mxr2 is very low. In addition, human mitochondrial MsrA and yeast cytosolic Mxr1 fail to bind to GrpEL1 irrespective of H_2_O_2_ treatment (Fig. 5C and E). To ascertain the binding activity of MsrA, we repeated the Ni-NTA pull down assay using MRP6, a known substrate of Msrs instead of GrpEL1. We observe a specific interaction of MsrA with MRP6 pre-treated with H_2_O_2_ (Fig. 5F). Our results provide evidence that Met R specific reductases either from yeast or human are able to specifically interact with GrpEL1 in an oxidation dependent manner potentially indicating a physiological role for the binding.

### Reduction of hGrpEL1-Met SO at Met146 by R type enzymes

**Figure 6:**
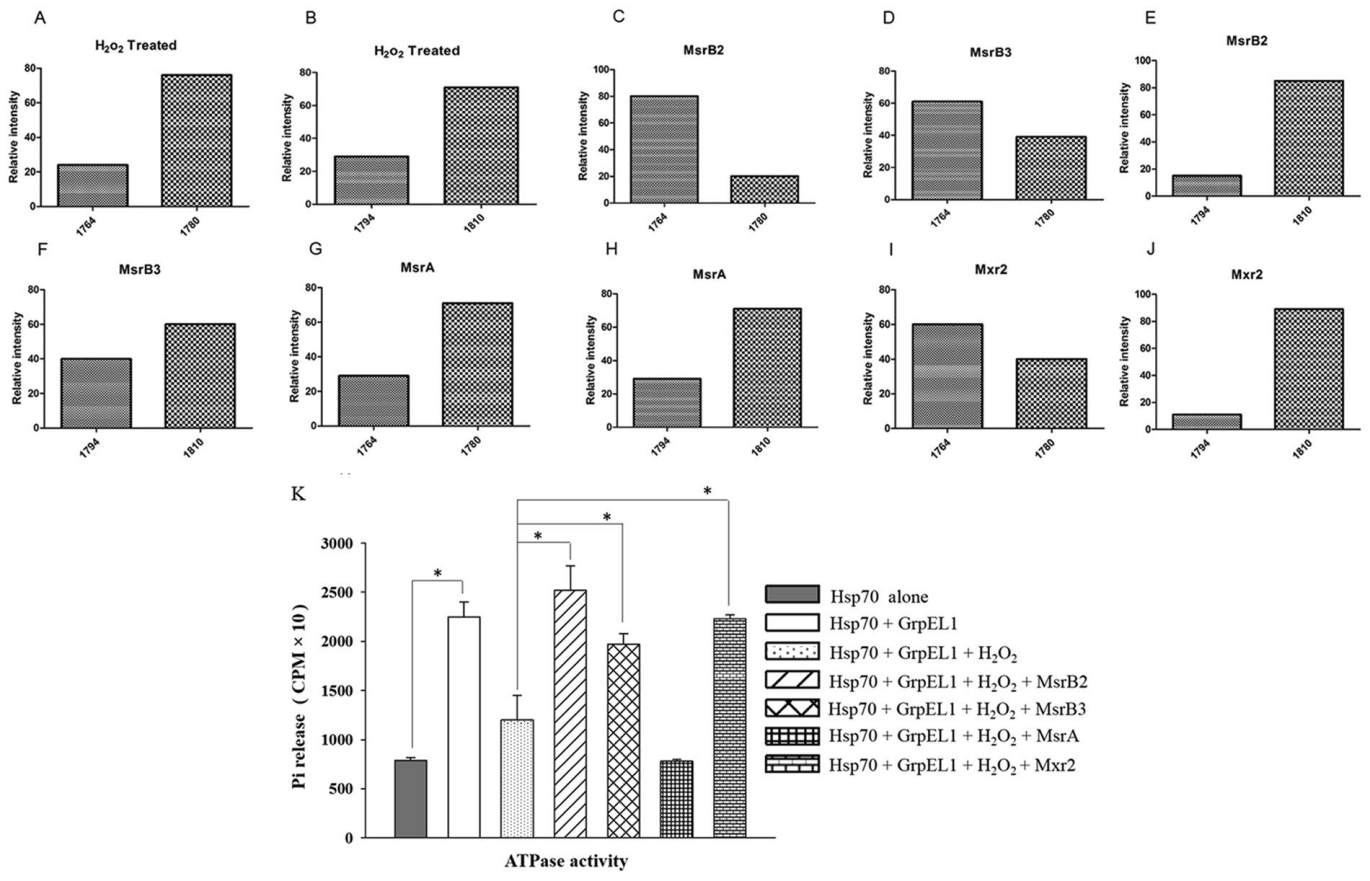
Specific reduction and re-activation of oxidized hGrpEL1 proteins by R type Msr enzymes. Oxidized hGrpEL1 (20 µg) protein was treated with different Msr proteins in a reaction buffer and incubated for 2 hours. The reactants were resolved on SDS-PAGE and the gel Coomassie stained. hGrpEL1 protein bands were excised, treated with trypsin and the resultant peptides were analyzed by MALDI-TOF followed by relative quantification of methionine containing peptide peaks. Percentage of oxidized and un-oxidized forms of Met146 and Met44 containing peptides were calculated from the MALDI spectra and shown here as relative intensity. The relative levels of total oxidized and un-oxidized M146 and M46 peptides were calculated from three independent experiments and the average of total oxidized and un-oxidized peptide are shown. (**A**,**B**) Relative intensity of oxidized and un-oxidized Met146 (**A**) and Met44 (**B**) peptides when hGrpEL1 protein was oxidized with H_2_O_2_ are shown. (**C**–**F**) Percentage of oxidized and un-oxidized Met146 (**C**,**D**,**G**,**I**) and Met44 (**E**,**F**,**H** and **J**) peptides when oxidized hGrpEL1 protein was reduced with MsrB2 (**C**,**E**), MsrB3 (**D**,**F**), MsrA (**G**,**H**) and Mxr2 (**I**,**J**). (**K**) Single turnover ATPase activity of Hsp70/Ssc1. Ssc1ATPase activity was performed as described in the Methods section. hGrpEL1 protein was treated with or without H_2_O_2_ (5 mM) for 2 hours, followed by incubation with different Msr enzymes for 1 hr and the ATPase activity of Hsp70/Ssc1 was analyzed as described in the Methods section. ATP hydrolysis was monitored by analyzing the released ^32^Pi using scintillation counter and a graph plotted using values obtained from three experiments. The values are mean values obtained from three technical replicates. Each value is mean ± SE (n = 3), *p ≤ 0.05.

*In vitro* pull down assays described above clearly demonstrate that methionine sulfoxide R reductases bind with relatively greater affinity to the oxidized form of GrpEL1/Mge1 than to its reduced form. In contrast, the S type of methionine sulfoxide reductases are unable to interact with either form of GrpEL1/Mge1. To understand if the interaction between the R type Msrs and the oxidized form of GrpEL1 have functional consequences, we utilized MALDI/TOF analysis to examine the oxidized state of GrpEL1 in the presence of Msrs. As observed earlier, majority of the recombinant GrpEL1 protein is oxidized with H_2_O_2_ treatment. The oxidation state is reflected by the appearance of peptides with masses 1780 Da and 1810 Da that correspond to oxidized Met146 and Met44 peptides respectively (Fig. 6A and B). Strikingly, when oxidized GrpEL1 was further incubated with the R type of reductases (MsrB2 or MsrB3), we observe a selective reduction in the percentage of oxidized 1780 Da peptide with a concomitant increase in the reduced Met1764 Da peptide (Fig. 6C and D). However, there is no efficient reduction in the 1810 Da peptide that harbors the oxidized Met44 amino acid (Fig. 6E and F). MsrA had no effect on the MALDI spectrum of oxidized GrpEL1 (Fig. 6G and H). Consistent with mammalian R type Msrs, yeast Mxr2 is able to efficiently reduce oxidized GrpEL1 at Met146 and not Met44 (Fig. 6I and J), a site that corresponds to Met155 in its canonical substrate, Mge1. Taken together, our results show that human MsrB3 and MsrB2 along with yeast Mxr2 can efficiently reduce oxidized GrpEL1 at Met146 position but not at Met44 position *in vitro*.

Next, we wished to test the functional relevance of oxidized GrpEL1 reduced by MsrB at Met146 in an ATPase stimulating activity of yHsp70/Ssc1. The results from this assay will also allow us to differentiate the effect of Met44/Met146 oxidation in the influence of ATPase stimulating activity by GrpEL1. We performed ATPase stimulating activity of GrpEL1 on yHsp70/Ssc1 as described in the Methods section. We pre-treated recombinant GrpEL1 with or without H_2_O_2_ prior to incubating it with yeast or mammalian Msrs. After the incubation step with Msrs, the ATPase stimulating activity was measured as described in Methods. yHsp70 alone exhibits a low intrinsic ATPase activity (Fig. 6K). Addition of recombinant GrpEL1 stimulates ATPase activity of yHsp70 by four folds while oxidized GrpEL1 does not have any significant effect. Prior incubation of oxidized GrpEL1 with R type of Msr’s (MsrB2, MsrB3 and yMxr2) restores the ATPase stimulating activity of GrpEL1 (Fig. 6K). However, pretreatment of oxidized GrpEL1 with S epimer reducing enzyme, MsrA has no effect in augmenting the ATPase activity of yHsp70 by GrpEL1. Our studies thus far, strongly suggest that the observed binding between oxidized GrpEL1 and the R type Msrs irrespective of their origin have a functional consequence as evident from the restoration of the ATPase stimulating activity of a ‘reduced’ GrpEL1 by Msr enzymes.

### *hGRPEL1 M146L* rescues the oxidative sensitive phenotype of yeast *MXR2* deletion

**Figure 7:**
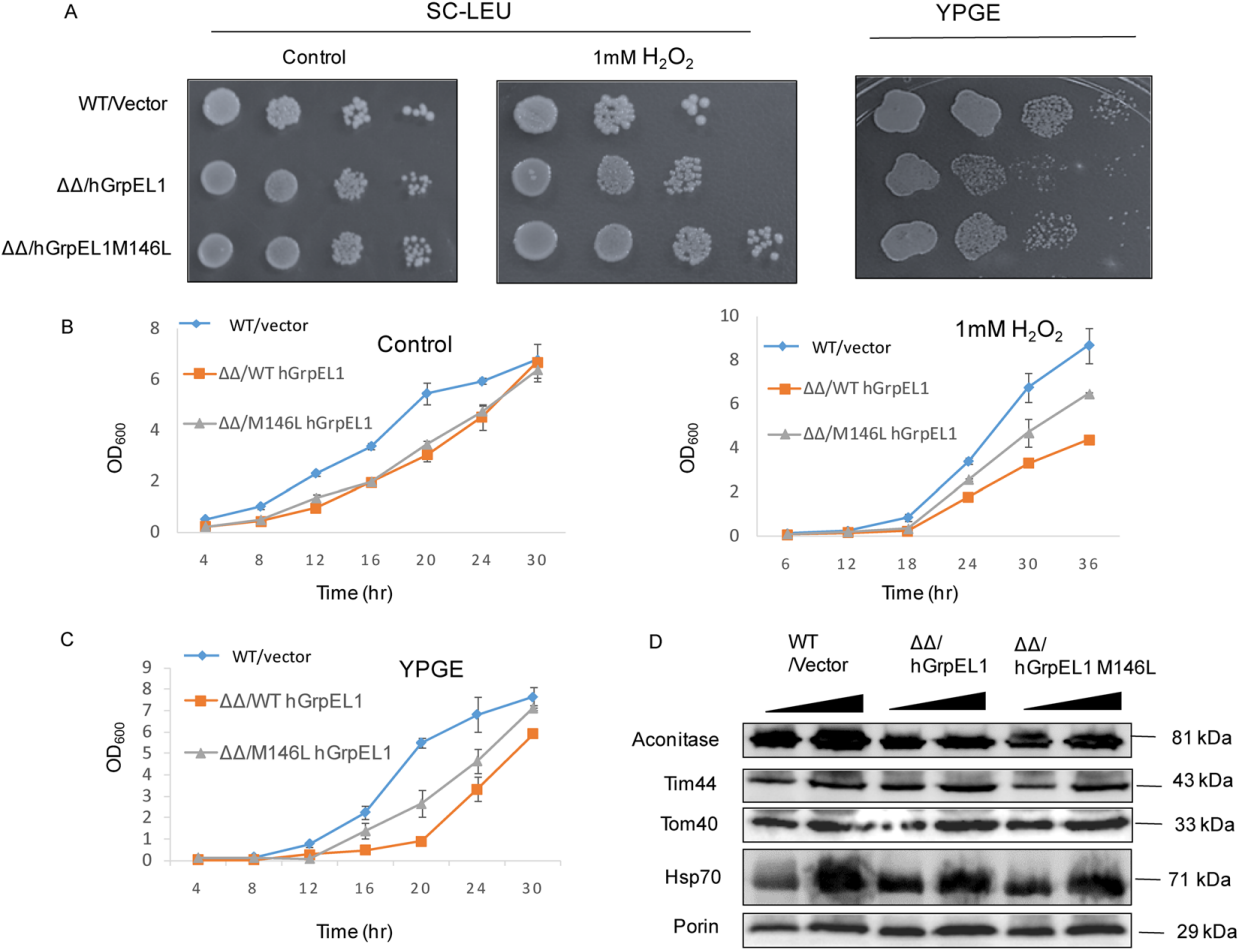
GrpEL1 M146L rescues the growth defect phenotype of *mxr2*Δ during oxidative stress. Growth phenotype of yeast strains expressing WT hGrpEL1 and hGrpEL1 M146L in a *mxr2*Δ background along with vector control were monitored in the presence and absence of H_2_O_2_ on minimal media and non-fermentable carbon sources. Overnight grown cultures were normalized to OD_600_ 1.0 and 10 μl of serially diluted samples were spotted on SC-Leu plated with or without H_2_O_2_ (left and middle panels of (**A**) and on YPGE plates (right panel of A). Liquid growth assay was performed in SC-Leu +/−H_2_O_2_ (**B**) and YPGE (**C**) by taking OD_600_ at different time points. A growth curve was plotted using the values obtained from three different replicates. (**D**) 50 and 100 μg of mitochondria isolated from wild-type, hGrpEL1 and hGrpEL1-M146L mutant were analyzed by SDS–PAGE, western blotted and probed with antibodies against specific mitochondrial proteins as shown.

Ectopically expressed Mge1 is able to complement the chromosomal deletion of *MGE1*. We have shown previously that an oxidative resistant mutant, *MGE1 M155L* is able to complement both *MGE1* and *MXR2* deletions under oxidative stress^15,19^. To probe if GrpEL1 M146L can functionally mimic Mge1 M155L mutant, we generated yeast strains that ectopically express either wild type *hGRPEL1* or *hGRPEL1 M146L* in a *mxr2*Δ and *mge1*Δ background as described in the Methods section. The growth phenotype of strains expressing either *hGRPEL1* or *h GRPEL1 M146L* were compared to their parent wild type strain with vector alone as control in the presence and absence of H_2_O_2_ on SC-Leu plates. Yeast strains expressing wild type and mutant GrpEL1 grew normally in the absence of H_2_O_2_ while GrpEL1 M146L mutant displayed better growth on minimal plates in presence of H_2_O_2_ (Fig. 7A). Similar growth resistant phenotype of hGrpEL1 M146L was observed in liquid cultures with H_2_O_2_ (Fig. 7B). These results indicate GrpEL1 is sensitive to oxidation *in vivo* under normal physiological conditions. We have shown earlier that *mxr2*Δ strain fails to grow on non-fermentable carbon sources while *MGE1 M155L* can complement the growth defect. Similarly, we find that *GRPEL1 M146L* is able to grow on non-fermentable carbon source when compared to the strain expressing WT *GRPEL1* in *mxr2*Δ strain background (Fig. 7A right panel and 7C). Further, we have observed no change in the steady state levels of WT GrpEL1, GrpEL1 M146L and other mitochondrial proteins in yeast strains expressing either WT hGrpEL1 or hGrpELl M146L mutant in *mxr2*Δ background (Fig. 7D, Supplementary Figure S3). Together, our results provide compelling evidence to the existence of an evolutionarily conserved mechanism that is orchestrated by mitochondrial proteins Mxr2-Mge1-Ssc1 to regulate cellular redox homeostasis.

## Discussion

Molecular Chaperones are essential components of cellular protein homeostasis and belong to a highly conserved protein family. Most of the chaperones assist in protein folding by utilizing ATP as an energy source^10,23^. Certain chaperones display stress dependent expression and^24^ are also regulated by post translational modifications. Very few chaperones have been reported to be regulated through reversible oxidative modifications at cysteine or methionine residues. It has been shown that thermal stress induces the expression of Hsp33 gene and additionally regulates its activity through a redox mechanism by forming disulfide bond at its zinc binding site^25,26^. Bacterial Hsp70 undergoes reversible glutathionylation in response to thermal and oxidative stress^27^. In eukaryotes, oxidation of Cys residues within cytosolic Hsp70 and Hsp90 chaperones result in their inactivation and induction of Unfolded Protein Response (UPR)^24,28^. In addition, Met and Cys oxidation in the eye lens chaperone, α-Crystallins is thought to be one of the major causes of eye defects^29^.

Most of the mitochondrial matrix targeted proteins are nuclear encoded, translated in cytosol and imported into mitochondria as pre-proteins^30^. Hsp70/Ssc1 chaperone is a component of the import motor that plays an essential role in pulling the proteins across inner membrane into mitochondrial matrix. Hsp70/Ssc1 chaperone system is also required for mitochondrial proteostasis, iron sulfur cluster biogenesis and in mtDNA maintenance^31^. Mge1/GrpEL1 is a conserved essential subunit of Hsp70/Ssc1 machinery and is majorly required for the efficiency of ATPase cycle. Dimeric GrpEL1 structure has two long N-terminal α-helix regions, a small four-helix bundle region with antiparallel topological arrangement followed by a C-terminal β-sheet domain^32,33^. Several point mutations in the four helix bundle of *E.coli* GrpE and yeast Mge1 are known to cause severe growth defects and loss of Ssc1 dependent functions^15,34^. Methionine 155 in yMge1 and the corresponding 146 in hGrpEL1, both resident in the four helix bundle region, are sensitive to oxidative stress and are conserved across eukaryotes. Yeast Mxr2 interacts with Mge1 in an oxidative stress dependent manner and restores Mge1 dependent Hsp70/Ssc1 functions both *in vitro* and *in vivo*^15,19^. In this report, we show that hGrpEL1 modulates the ATPase activity of yHsp70/Ssc1 during oxidative stress (Fig. 2). In mammals, two isoforms of GrpEL (GrpEL1 and GrpEL2) are present in the mitochondria and their presence is ubiquitous. Our results show that hGrpEL1 but not hGrpEL2 is able to complement yMGE1 deletion indicating that it is probably playing a role similar to Mge1 in regulating the activity of Hsp70/Ssc1 chaperone (Fig. 3 and unpublished results). Interestingly, hGrpEL2 does not contain methionine corresponding to the conserved Met155 position of yeast Mge1. Oxidation resistant mutant, hGrpEL1 M146L is able to rescue the growth defect associated with *mxr2*Δ strain in presence of H_2_O_2_ indicating the conserved role of GrpEL1 during oxidative stress (Fig. 7).

Msrs act as antioxidants to regulate various biological processes. Msrs reduce Met-R-SO and Met-S-SO oxidized derivatives of methionine. They are evolutionarily conserved, and human mitochondria contain MsrA, MsrB2 and MsrB3 isoforms. MsrBs display a preference to proteins that have Met R SO oxidized form rather than Met-S-SO^5^. Our previous and current *in vitro* data suggest that oxidized hGrpEL1/Mge1is specifically reduced by R epimer reducing enzymes. Although both methionines in GrpE1 are oxidized *in vitro*, we find Met44 to be pre-oxidized during most of our experimental conditions. This may be due to auto-oxidation that has been reported earlier^35^. Intriguingly, we have not observed a reduction of oxidized Met44. Met44 is present in the un-structured tails of GrpEL1 that is part of the non-conserved region and not essential for GrpEL1 dimerization nor for stimulating Hsp70/Ssc1 ATPase activity. MsrB isoform shows preference towards M146-SO over Met44-SO (Figs 5 and 6) in reducing the sulfoxide. Additionally, only R type of human *MSR* isoforms can complement the loss of *yMXR2* under oxidative conditions. Based on the above results, we believe that the conserved oxidized methionine in Mge1 and GrpEL1 is most likely to be the R epimer form. Besides mitochondria, MsrB3 is also localized to the ER in mammals^36^. Interestingly, we find that *MSRB3* lacking the upstream Su9-MTS sequence fails to complement *MXR2* deletion under oxidative stress (unpublished results). This shows that ER targeted MxrB3 has no apparent role in mitochondria.

Further, we find that *hGRPEL1 M146L* mutant complements the slow growth phenotype of *MXR2* deleted cells (Fig. 7). Curiously, we find that wild type strain grows significantly much faster than cells devoid of *MXR2* but harboring hGrpEL1 M146L in liquid cultures when compared to plate based assay. It has been shown that good aeration exacerbates the growth and viability defect caused by deletion of superoxide dismutase (*SOD*), while this difference from wild type strain is mitigated under low aeration^37^. Good aeration increases the release of ROS in mitochondria. We believe that this same effect is seen in the case of absence of *MXR2*. In liquid cultures where there is good aeration, the effect of *MXR2* deletion is exacerbated. In contrast, in a plate assay that causes lower aeration, hGrpEL1 M146 mutant even in absence of MXR2 exhibits better growth characteristics. Additionally, cells harboring WT hGrpEL1 exhibit a growth phenotype that is akin to cells harboring hGrpEL1 M146L at later time points in liquid culture. This may due to the diminishing amount of H_2_O_2_ with time.

Mitochondria accounts for 90% of total cellular ROS. Physiological levels of ROS regulate protein functions, however, increased ROS can damage various biomolecules. Excessive buildup of oxidized (reversible and irreversible) macromolecule aggregates promote aging^38^. Dysfunctional mitochondria, environmental factors or decreased antioxidant capacity of cell can contribute to imbalances in ROS production^1,39,40^. Methionine oxidation, loss of Msrs and increased mitochondrial ROS have been implicated in several neurological disorders like Parkinson’s and Alzheimer’s diseases including aging. We hypothesize that GrpEL1 oxidation might be playing a key role in Msr associated pathologies. Age dependent accumulation of ROS or decreased antioxidant property or altered Msr activities can lead to increased Met SO of GrpEL1. The cascading effects of Met oxidation of Mge1/GrpEL1 include defective chaperone system and altered protein homeostasis that further aggravate several mitochondrial associated disease conditions. Mitochondrial hGrpEL1 perhaps acts like a redox sensor that transduces appropriate signals for mitochondrial protein homeostasis.

## Methods

### Plasmid construction

Plasmids *MSRA*, *MSRB2*, *MSRB3* were purchased from DNASU plasmid repository. *MSRA* gene was amplified with primers *MSRA*_Fwd1 (5′-CCCA GAATTC ACC ATG GCT GTA TTT GGA ATG-3′) and *MSRA*_Rev1 (5′-CCCC CTC GAG TTT TTT AAT ACC CAC TGG GCA-3′) and cloned into a pET28a + vector to generate pNB638. *MSRB2* and *MSRB3* genes were amplified using primer pairs *MSRB2*_Fwd2 (5′-AAAA CCATG GCG CGG CTC CTC TGG-3′), *MSRB2*_Rev2 (5′-AAAA GGATCC ACC ATG GCG CGG CTC CTC TGG-3′) and *MSRB3*_Fwd3 (5′-AAAA CCATG GGC TCT GCA TTC AAC CTG CTG -3′), *MSRB3*_Rev3 (5′- CCCC AAG CTT CTC CGC TTT GTC TGC CTG -3′) and cloned into pET28a + to generate pNB640 and pNB648 respectively. For *in vivo* expression in yeast, human *MSR* genes were sub-cloned into vector downstream of SU9 MTS and with C-terminal FLAG epitope. SU9 MTS was amplified from pNB03 using primers Su9-Fwd4 (5′AACC ACTAGT ACC ATG GCC TCC ACT CGT GTC3′) and Su9-Rev4 (5′AAAA GGATCC GGA AGA GTA GGC GCG CTT3′) with restriction sites SpeI/BamHI and cloned into pTEF-URA to create PNB669. *MSRA* and *MSRB3* were amplified with primer pairs *MSRA*-Fwd5 (5′-CCCA GGATCC ATG GCT GTA TTT GGA ATG -3′), *MSRA*-Rev (5′-CCCC AAG CTT TTT TTT AAT ACC CAC TGG-3′) and *MSRB3*-Fwd7 (5′-AAAA GGATCC ACC ATG TCT GCA TTC AAC CTG-3) and *MSRB3*-REV3. The amplified products were cloned into pNB543 as BamHI - HindIII fragments to generate pNB639 and pNB643 containing *MSRA* and *MSRB3* respectively. Since *MSRB2* contains internal BamHI site, we amplified full length gene with primers *MSRB2*-Fwd2 and *MSRB2*-Rev2. Amplified gene was digested with NcoI and HindIII and cloned into SpeI and HindIII predigested plasmid pNB475^19^ to express *MSRB2* full length protein with FLAG epitope. Similarly, *GRPEL1* WT was sub-cloned into pTEF LEU plasmid to generate plasmid pNB598 with Su9 MTS for yeast expression. Pet28a^+^ carrying *GRPEL1-M146L* (pNB555) was created by site-directed mutagenesis of *GRPEL1* present in pNB243 using primers *GRPEL1*_Fwd (5′GGG CTG GTC CTG ACT GAA GTC 3′) and *GRPEL1 Rev (5*′*-GAC TTC AGT CAG GAC CAG CCC-*3′*)*. The mutant *GRPEL1* was further amplified using pNB555 as template and sub-cloned into pTEF LEU plasmid to generate pNB605 with Su9-MTS.

### Protein expression and purification

Plasmids containing *His-MSRA*, *His-MSRB2*, *His-MSRB3* and *His-MRP6* genes were transformed into *E. coli* BL21 (DE3) Codon Plus (RIL) cells. Protein expression was induced with 1 mM IPTG and soluble proteins were further purified by using Ni-NTA talon affinity resin (GE Healthcare). Purified proteins were dialyzed in phosphate buffered saline (PBS) pH 7.2 with 5 mM β-ME. Similarly, *hGrpEL1* wild type protein was expressed, purified and dialyzed in PBS. yMxr2, yMge1 and Hsp70/Ssc1 proteins were expressed and purified as described earlier^15,19^. All the purified proteins were separated on SDS-PAGE to assess the purity (Supplementary Figure S4).

### Yeast strain construction

Yeast strain yNB65 is deleted for chromosomal *MGE1* but has *MGE1* expressed ectopically from a high copy URA3 plasmid (14). yNB158 and yNB159 were created by plasmid shuffling pNB598 (*hGRPEL1* in a high copy LEU2 plasmid) and pNB605 (*hGRPEL1 M146L* high copy LEU2) respectively into yNB65 and evicting the URA3 plasmid by growing the transformants on 5 FOA. The transformants were further confirmed by checking the loss of viability on 5-FOA and growth on SC-URA plates (Supplementary Fig. 1), by PCR for *MGE1* deletion and by immunoblot analysis for hGrpEL1. yNB126 is deleted for chromosomal copies of *MXR2* and *MGE1* while ectopically expressing *MGE1* from a high copy URA3 plasmid^15^. The URA3 plasmid in yNB126 was evicted by growing the PNB598 and PNB605 transformants on 5 FOA to generate yNB138 and yNB139 expressing *hGRPEL1* and *hGRPEL1 M146L* respectively. yNB117 is deleted for chromosomal *MXR2*^19^ and was used to generate yNB145, yNB146 and yNB147 by transforming it with URA3 high copy plasmids pNB639 (*MSRA*), pNB641 (*MSRB3*) and pNB645 (*MSRB2*) respectively. The strains were selected by growth on SC-URA plates and confirmed by immunoblot analysis with anti-FLAG antibody.

### Yeast media and Growth assay

Standard conditions were used for culturing and maintaining of yeast strains. To evict URA3 plasmid, yeast strains were plated on SC medium containing 5 FOA (0.67% SC-URA, 2% dextrose, 50 μg/ml uracil, 2% agar and 0.1% 5-FOA). For performing growth assays, yeast strains were freshly streaked onto a YPD plates and the resultant colonies were grown in YPD medium overnight. These cultures were normalized to OD_600_ 1.0 and subjected to a ten-fold serial dilution. 5 µl from each dilution was spotted on SD-Leu plates with or without 1 mM H_2_O_2_ and on YPGE plates (1% yeast extract. 2% peptone, 3% glycerol and 2% ethanol). Images were taken after 2 days of incubation in case of SD plates or 3–4 days in case of YPGE plates at 30 °C.

### *In vitro* interaction assay

100 μg Mitochondrial lysate from the strains expressing FLAG-MsrA, FLAG-MsrB2, FLAG-MsrB3, FLAG-Mxr2 or 100 μg cytosol lysate from the strain expressing FLAG-Mxr1 were used for *in vitro* interaction studies with or without H_2_O_2_ treated 10 μg of His-hGrpEL1 or His-Mrp6 proteins. His-hGrpEL1 and His-Mrp6 were allowed to bind to Ni-NTA beads and the lysates were passed through. Ni-NTA beads were washed 3 times with 10 mM imidazole buffer. The beads were collected, suspending in SDS sample buffer and separated on SDS-PAGE. The gels were transferred for immunoblot analysis.

### *In vitro* reduction of oxidized hGrpEL1 by Msrs

Enzymatic activities of Msrs were initially checked on methionine rich Mrp6 protein pre-treated with H_2_O_2_ followed by reduction in a Trx coupled system as described^41^. Oxidized GrpEL1 was incubated with Msr proteins in a reaction buffer containing 50 mM sodium phosphate, 50 mM sodium chloride and 10 mM DTT and separated on SDS-PAGE. The gel was Coomassie stained and the GrpEL1 bands were excised and subjected to trypsin digestion prior to mass spectrometric analysis.

### Hsp70/Ssc1 single turnover ATPase assay

Hsp70/Ssc1 ATPase activity assay was performed as described earlier^15^ with minor modifications. yMge1 and hGrpEL1 proteins were pre-treated with or without 5 mM H_2_O_2_ and dialyzed. 5 μg of yMge1 or hGrpEL1 were added to the reaction buffer (50 mM HEPES/ KOH, pH 7.2, 5 mM MgCl_2_, and 100 mM KCl) containing 2 µg of yHsp70/Ssc1 and 0.05 mM [γ- ^32^P] ATP (3000 Ci/mmol). The effect of Msr on Hsp70/Ssc1 ATPase activity *via* GrpEL1 was also monitored by incubating oxidized hGrpEL1 with 5 μg of Msr protein in presence of 10 mM DTT for 30 min prior to the Hsp70/Ssc1 assay. The amount of radioactive inorganic phosphate (pi) released after 5 min was measured in a scintillation counter as described^15^. Data was analyzed using Jandel scientific sigma software by one way ANOVA followed by post hoc Duncan’s test for multiple comparion.

### Mitochondria Isolation

Isolation of mitochondria was performed as described earlier^42,43^. Briefly, yeast strains expressing Flag-MsrB2, Flag-Su9-MsrB3 and Flag-Su9-MsrA were grown overnight in 2% lactate medium. Cells were centrifuged at 5000 rpm and washed with 100 mM Tris-SO_4_ pH 9.4 buffer followed by lysis with lyticase (Sigma- L2524) in 1.2 M sorbitol and 20 mM phosphate buffer pH 7.0. The lysed cells were homogenized in SEM buffer (250 mM Sucrose, 1 mM EDTA, 10 mM MOPS/KOH pH 7.2) and centrifuged at 3500 rpm. The supernatant was collected and centrifuged at 10000 rpm. The resultant mitochondrial pellet was washed twice and suspended in SEM buffer and stored at −80 °C. The cytosolic fraction from yeast strain expressing Flag-Mxr1 was obtained by following the above procedure except that in the last step, instead of mitochondrial pellet, the post-mitochondrial fraction/supernatant is taken and stored at −20 °C.

### MALDI studies

Mass spectrometry of hGrpEL1 proteins was performed as described earlier^19^. Briefly, 20 mM H_2_O_2_ treated 20 µg of hGrpEL1 was incubated with individual 10 µg of Msr proteins in a reaction buffer. The sample was incubated for 2 hours followed by separation of proteins on SDS-PAGE. The hGrpEL1 protein bands were excised from gel and digested with trypsin followed by mass spectrometric analysis. Relative levels of peptides of interest were quantified and plotted. The intensity of the peaks are taken into account while plotting the graph of oxidized and un-oxidized peptides.

### Antibodies and Statistical analysis

Mge1 and Tim44 antibodies were raised in-house^15^. Hsp70/Ssc1, porin, Aconitase and Tom40 antibodies are kind gifts from Prof. Debkumar Pain lab (Rutgers University). Glycerol kinase and Mrp6 antibodies were kind gifts from Prof.Avadhani (University of Pennsylvania) and from Prof. Gladyshev (Harvard University) respectively. Flag (F3165) and His (SAB1306084) antibodies were obtained from Sigma, Santa Cruz respectively. All the experiments were performed minimum three times.

## Electronic supplementary material

Supplementary data
