# Supplementary material for: A conserved R type Methionine Sulfoxide Reductase reverses oxidized GrpEL1/Mge1 to regulate Hsp70 chaperone cycle
Source: Sci Rep. 2018 Feb 9;8:2716. doi: 10.1038/s41598-018-21083-9 (PMC5807549; doi:10.1038/s41598-018-21083-9)
Supplement: Supplementary file 1 — Supplementary data [file 41598_2018_21083_MOESM1_ESM.pdf]

A conserved R type Methionine Sulfoxide reductase reverses oxidized GrpEL1/Mge1 to regulate Hsp70 chaperone cycle

Praveen Kumar Allu, Yerranna Boggula, Srinivasu Karri, Adinarayana Marada, Thanuja Krishnamoorthy and Naresh Babu V Sepuri*

From the Department of Biochemistry, School of Life Sciences, University of Hyderabad, Gachibowli, Hyderabad 500046, India.

* Correspondence: nareshuohyd@gmail.com or nbvssl@uohyd.ernet.in, Tel: 91-40-23134531

Supplementary information

Figure S1: Shuffling of *hGRPEL1* wild type and mutant plasmids in yeast *MGE1* deletion background

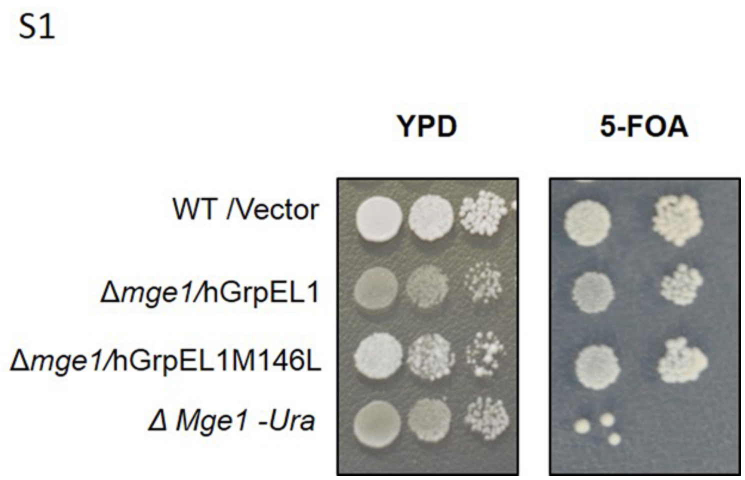

Figure S2: MALDI –TOF spectrums, Peptide Mass list of oxidized and un-oxidized hGrpEL1

A. Un-oxidized hGrpEL1 spectrum

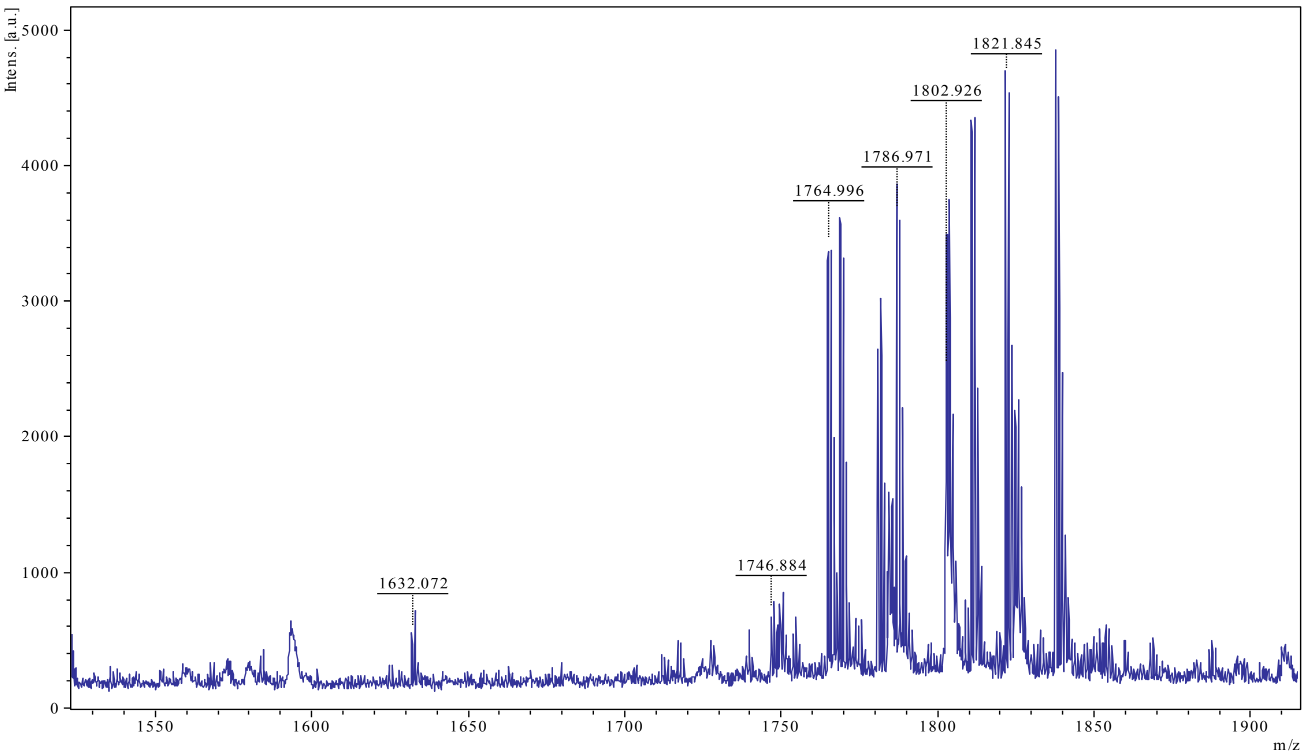

B. Un-oxidized hGrpEL1 Zoomed Spectrum

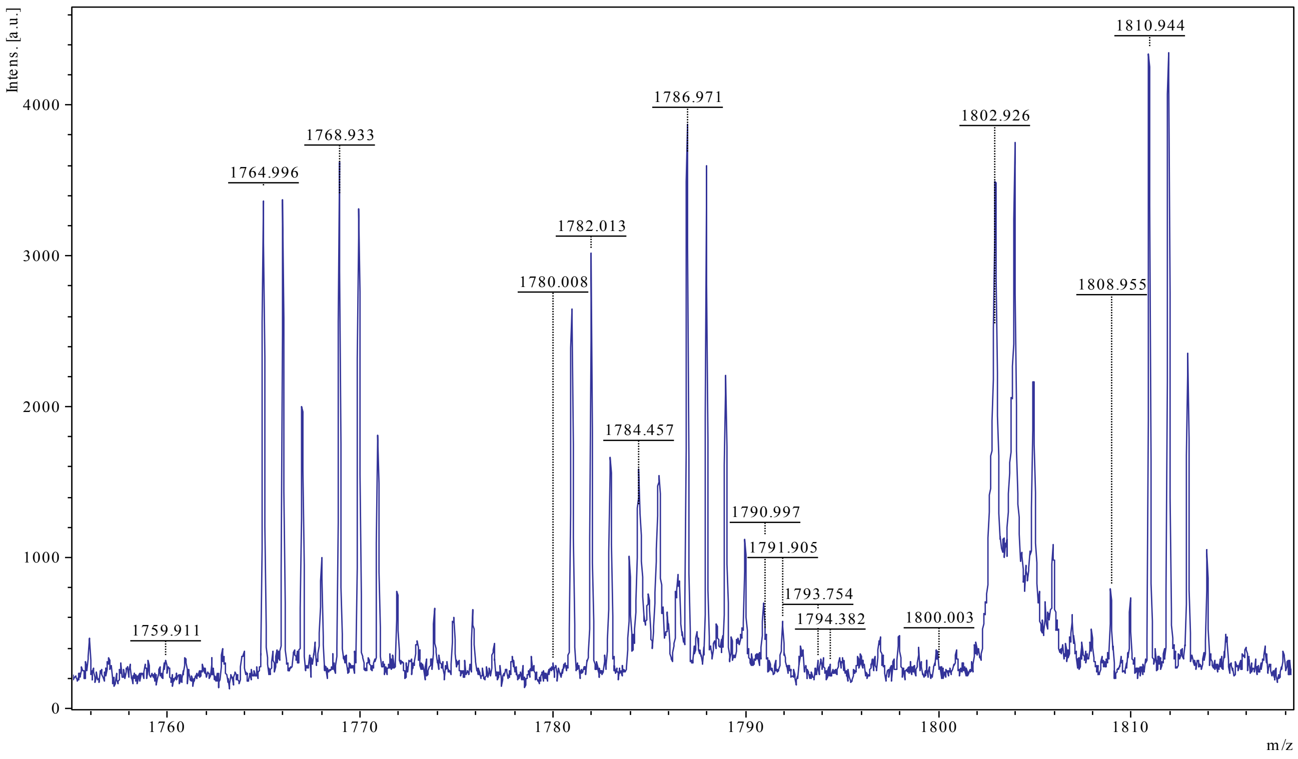

C. Oxidized hGrpEL1 Spectrum

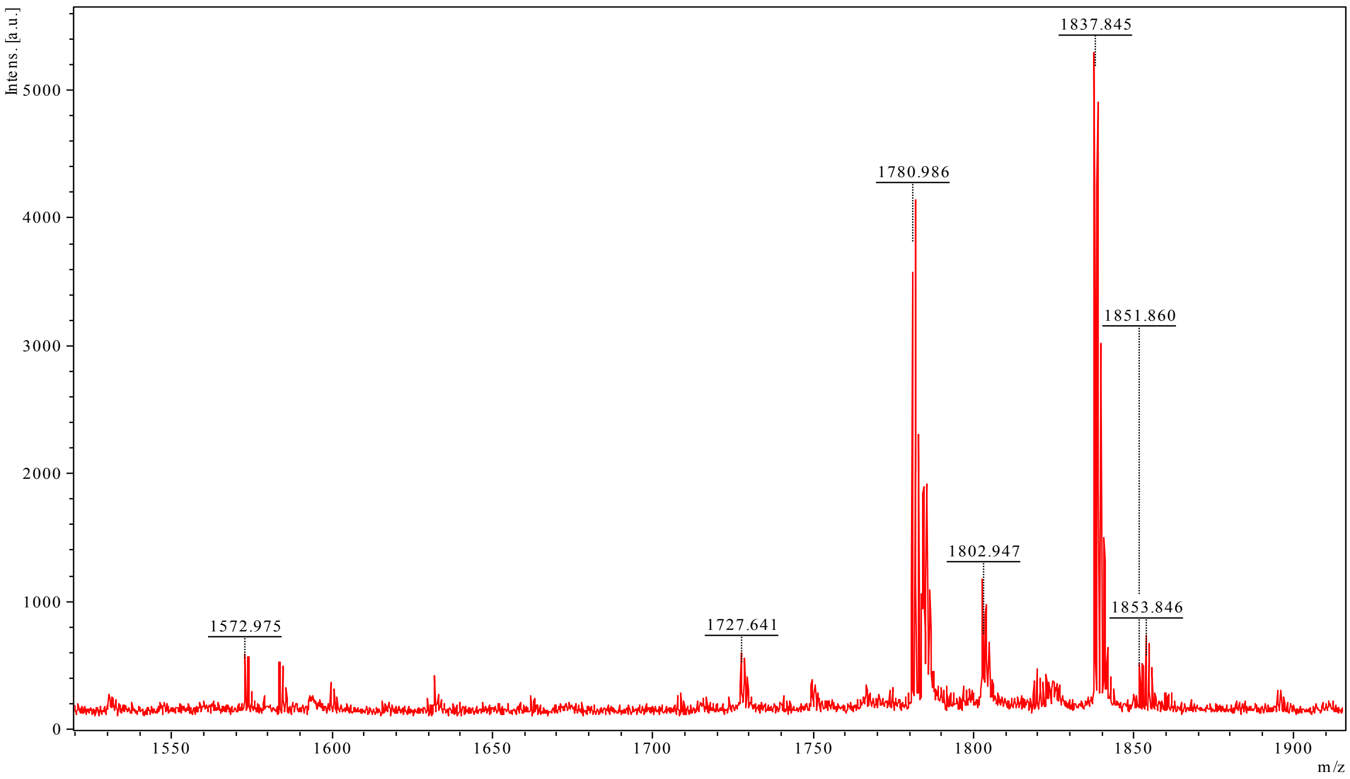

D. Oxidized hGrpEL1 Zoomed Spectrum

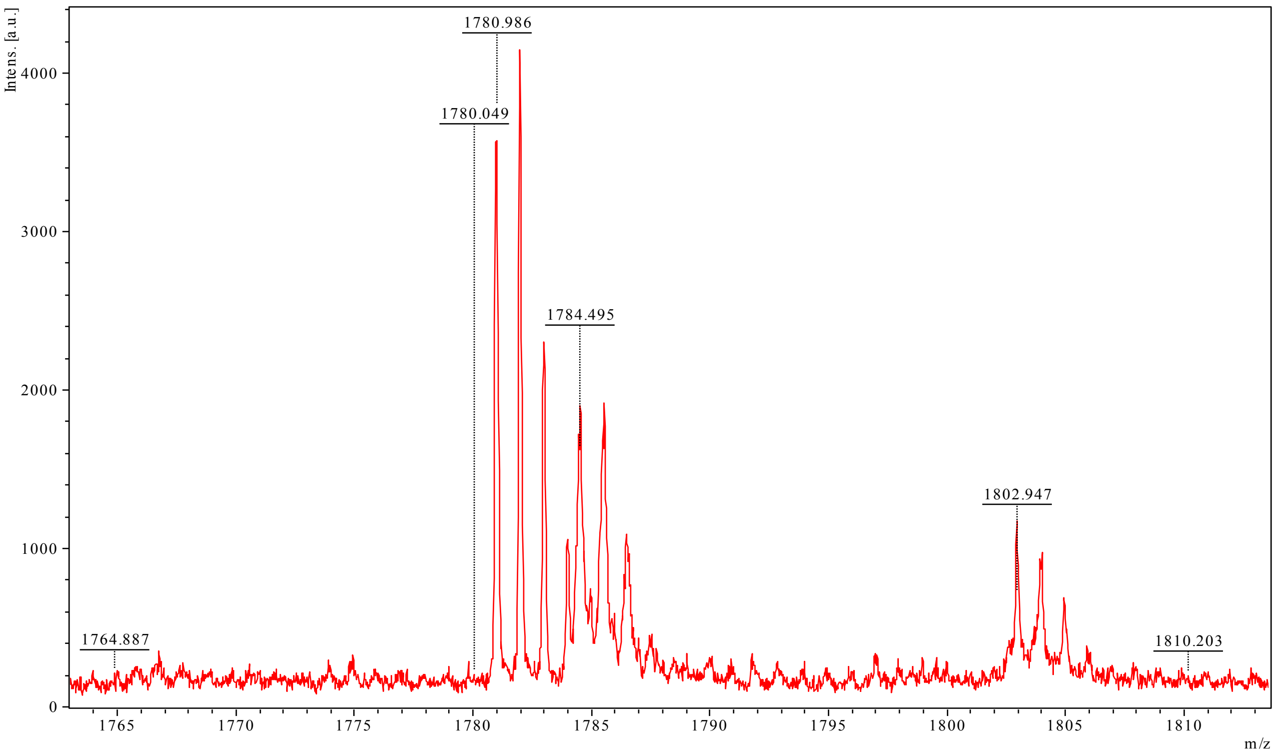

Figure S3: Steady state levels of WT hGrpEL1 and hGrpEL1-M146L in *mxr2*Δ strain

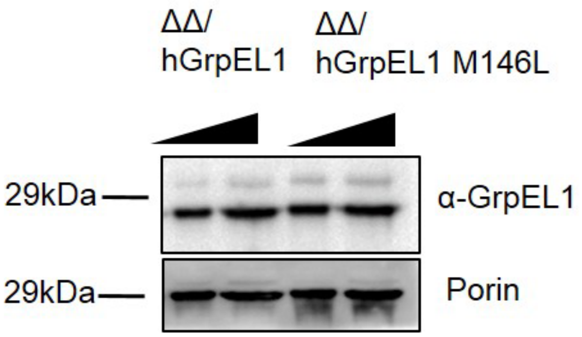

Figures S4: Purity of recombinant Mge1p, GrpEL1, and other enzymes

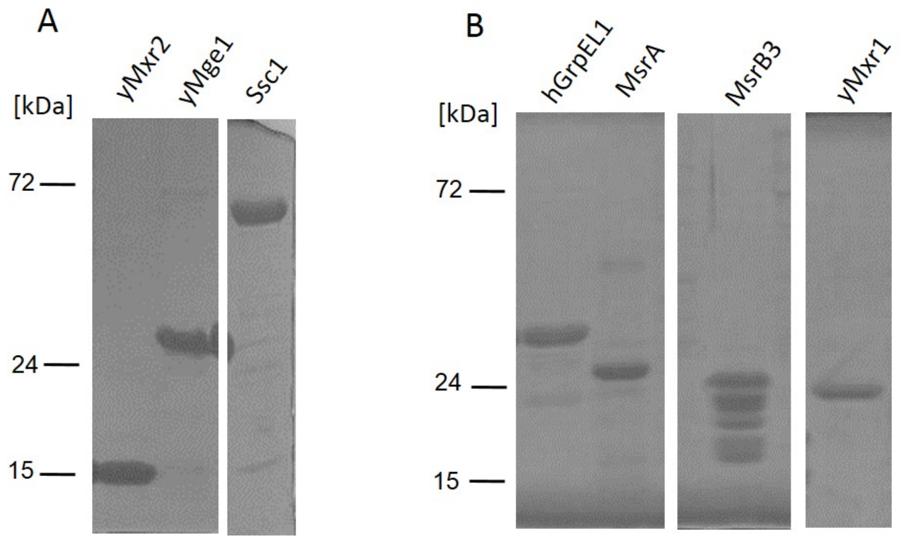

Figure S5: Full length blots of Figure 3C, 4B, 4C, 4D, 5A, 5B, 5C, 5D, 5E, 5F and 7D.

Figure 3C

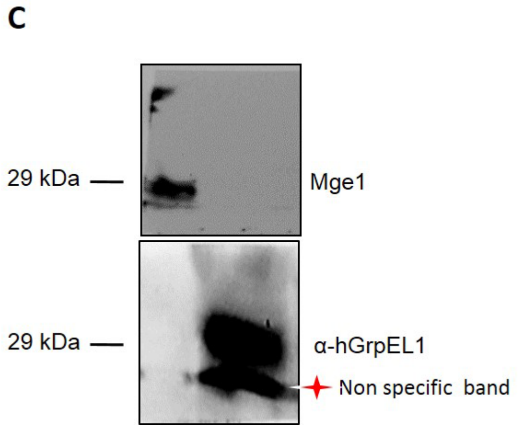

Figure 4

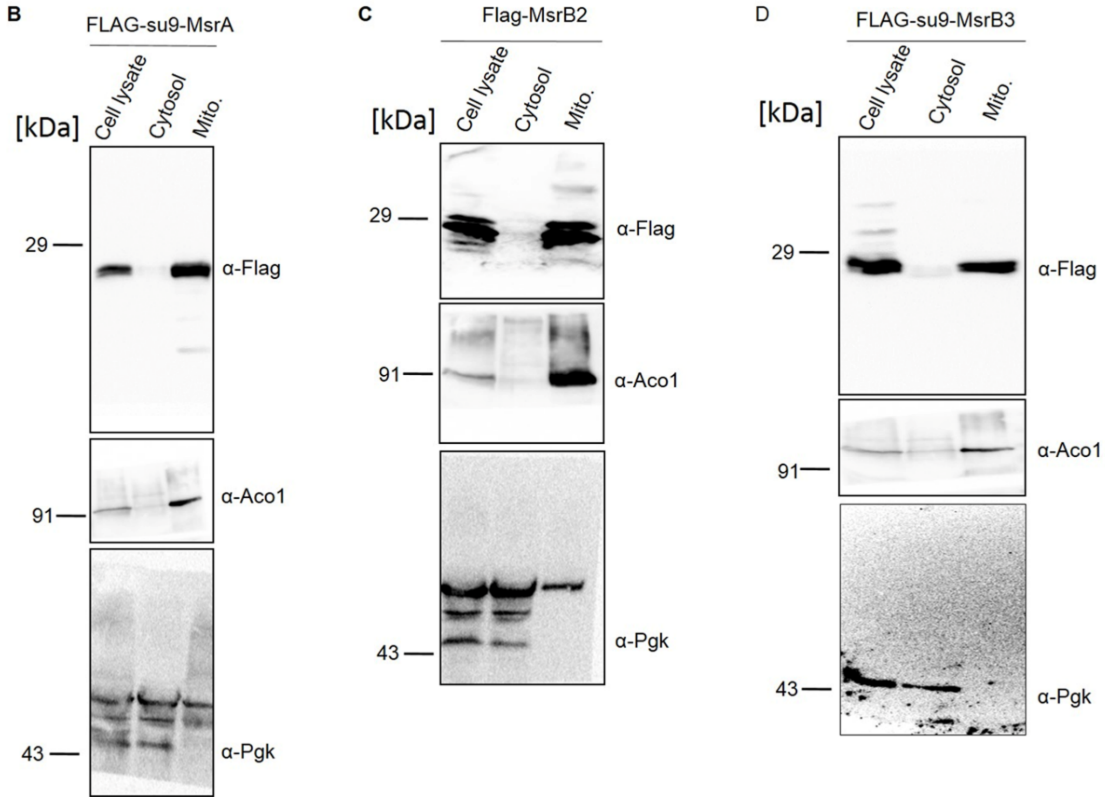

Figure 5

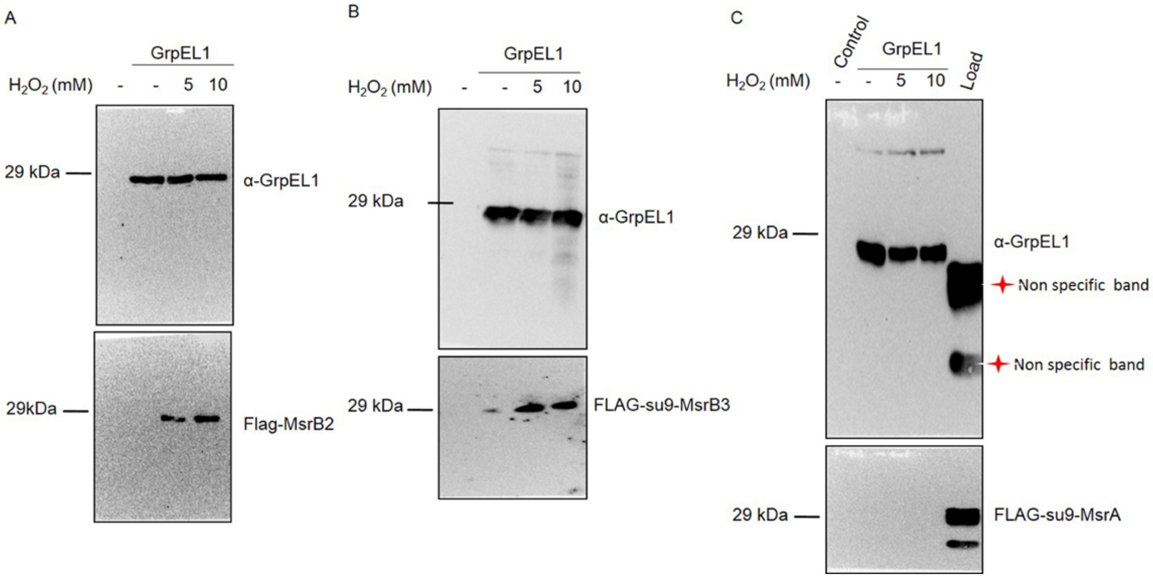

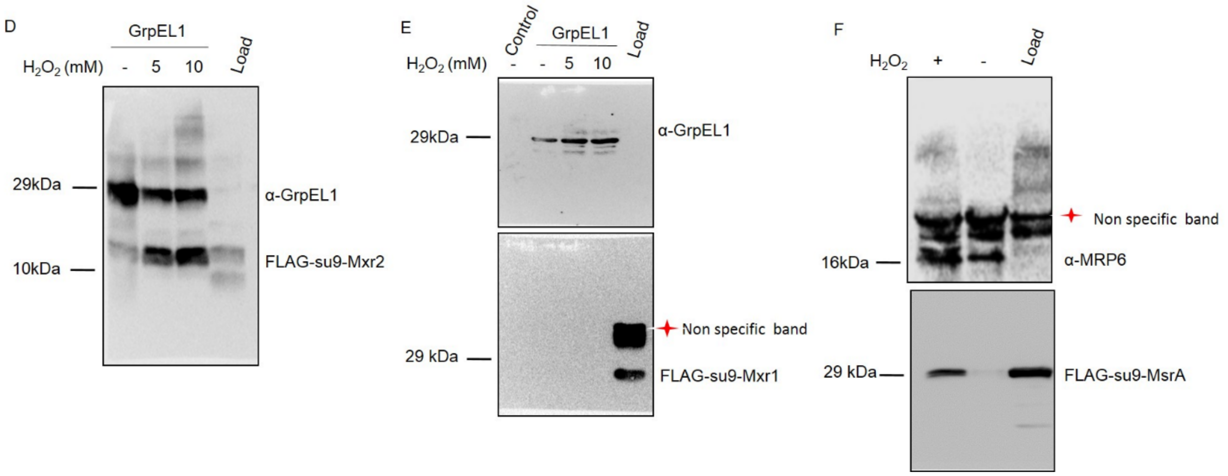
D
GrpEL1
Load
H2O2 (mM) - 5 10
29kDa
10kDa
α-GrpEL1
FLAG-su9-Mxr2
E
Control
GrpEL1
Load
H2O2 (mM) - - 5 10
29kDa
α-GrpEL1
29 kDa
Non specific band
FLAG-su9-Mxr1
F
H2O2 + -
Load
Non specific band
16kDa
α-MRP6
29 kDa
FLAG-su9-MsrA

Figure 7

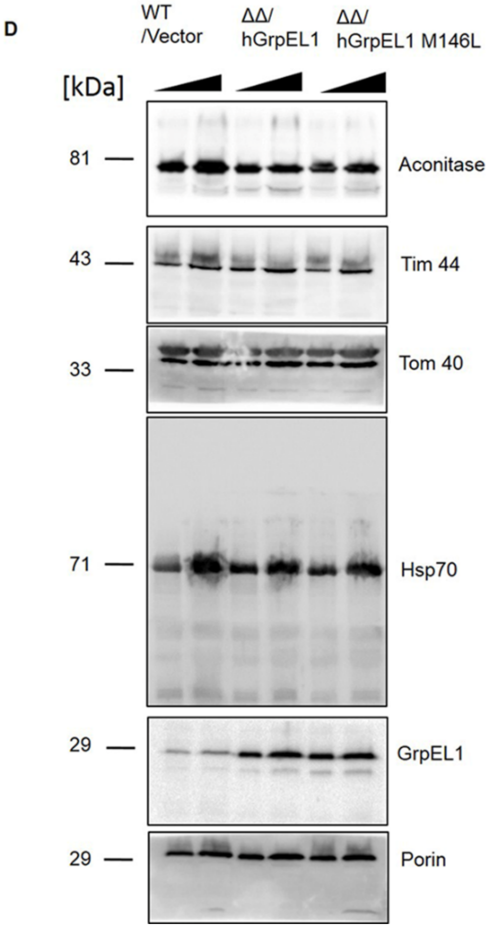
D
WT
/Vector
ΔΔ/
hGrpEL1
ΔΔ/
hGrpEL1 M146L
[kDa]
81
Aconitase
43
Tim 44
33
Tom 40
71
Hsp70
29
GrpEL1
29
Porin

Figure S1: Shuffling of hGrpEL1 wild type and mutant plasmids in yeast MGE1 deletion background

Yeast strains yNB186, yNB158, yNB159 expressing WT yMge1, hGrpEL1, and hGrpEL1 M146L respectively were grown overnight in YPD, normalized to OD_{600} 0.5 and 10 µl of each dilution were spotted on YPD and 5FOA plates.

Figure S2: MALDI-TOF spectrum of purified hGrpEL1 protein treated or untreated with H_2O_2

In vitro H_2O_2 treated or untreated purified hGrpEL1 protein was separated on SDS-PAGE and Coomassie stained. The excised hGrpEL1 protein was in gel digested with trypsin and analyzed by MALDI TOF Spectrum.

A & B represents the native or un-oxidized GrpEL1 MALDI-TOF while C & D represent oxidized spectrums. B and D spectrum are zoomed versions of A & B spectrum respectively. The mass of the peptides under consideration are shown in the spectrum (M146, 1764 Da; M146-S-O, 1780 Da; M44, 1794 Da; M44-S-O, 1810 Da).

Figure S3: Steady state levels of WT hGrpEL1 and hGrpEL1-M146L in *mxr2*Δ background

Increased amounts of mitochondria isolated from strains expressing wild-type hGrpEL1 and hGrpEL1-M146L mutant were separated on SDS–PAGE and immunoblotted with hGrpEL1 and porin antibodies.

Figure S4: Purity of recombinant proteins Mge1, Ssc1, GrpEL1, MsrB3, Mxr2, MxrA and Mxr1

A. Purified recombinant proteins of yeast Mge1, Mxr2, Ssc1 or (B) human GrpEL1, MsrA, MsrB3 and yMxr1 were resolved on 12% SDS–PAGE and Coomassie stained.

Figure S5: Full length blots.

Full length blots of Figure 3, 4, 5 and 7 probed with respective antibodies
